# Clozapine Intoxication in a Patient on Chronic Use With a Short-Term Noncompliance

**DOI:** 10.7759/cureus.16578

**Published:** 2021-07-23

**Authors:** Zoha Khan, Emily A Miller, Abdullah M Pervaiz

**Affiliations:** 1 Internal Medicine, University of Massachusetts Medical School - Baystate, Springfield, USA; 2 Internal Medicine, Tufts University School of Medicine, Boston, USA

**Keywords:** clozapine, intoxication, chronic, psychiatry, encephalopathy

## Abstract

Clozapine has been associated with various adverse effects at both subtherapeutic and standard doses. These adverse effects are most commonly seen during the initiation of therapy in treatment-naïve patients. However, reports of intoxication in patients with long-term use of clozapine are yet to be documented. We highlight the case of a 42-year-old gentleman who had been on long-term clozapine use and presented with an altered mental status after being resumed on full standard doses without careful titration of clozapine after a short period of medication noncompliance. His workup in the hospital was largely unremarkable, and his presentation was attributed to the resumption of clozapine without medication titration. This is the first case to demonstrate the necessity of careful titration regardless of previous treatment history and highlights that patients should be started on clozapine at low levels to avoid the possibility of acute intoxication.

## Introduction

Clozapine has been shown to cause various adverse effects including life-threatening neutropenia, agranulocytosis, and sedation at subtherapeutic as well as standard doses. These adverse effects are most commonly seen during the initiation of therapy in treatment-naïve patients. Previous cases have reported clozapine intoxication in treatment-naïve patients [1,2]. However, with regards to patients with long histories of clozapine use at standard doses, reports of intoxication have not been documented. Here, we present a case of clozapine intoxication in a patient with a long history of clozapine use.

## Case presentation

A 42-year-old man with a past medical history of schizophrenia was found outside a behavioral health facility and appeared groggy with slurring of speech. The patient was given naloxone by emergency medical services without improvement in his symptoms and was transported to the emergency department for further evaluation. On presentation, the patient was afebrile and hemodynamically stable. He was alert and oriented to person, place, and time; however, exhibited slurred speech and an inability to stay awake despite repeated stimulation. Lab work did not show electrolyte abnormalities, leukocytosis, or a deranged thyroid-stimulating hormone level (Table 1). Urine toxicology was negative for barbiturates, cannabinoids, cocaine, benzodiazepines, amphetamine, and opioids. His serum ethanol level was negative and his acetaminophen level was less than 5 µg/mL. His urinalysis was not indicative of infection. Clozapine level was noted to be 172 ng/mL (reference range: 350-650 ng/mL). Chest X-ray showed no acute cardiopulmonary abnormality. The patient reported that he was in his usual state of health the prior night when he restarted clozapine after approximately six weeks of medication noncompliance due to the inability to see his psychiatrist for a prescription. He stated that he took 300 mg of clozapine at the full dose the previous evening, and did not restart it with a taper. Medication history indicated that the patient had been prescribed haloperidol 5 mg daily and clozapine 100 mg in the morning and 300 mg in the evening for at least three years. He denied using any opioids or alcohol. He also reported no history of recent infection, shortness of breath, chest pain, or trauma. The following day, the patient was alert and oriented to person, place, and time, and was able to converse normally with no drowsiness or somnolence. He was evaluated by the psychiatry service, and his altered mental status was attributed to the resumption of clozapine at full dose after a period of medication noncompliance. The patient was restarted on clozapine at 25 mg twice daily with plans to increase slowly to therapeutic doses in the outpatient setting.

**Table 1:** Lab workup obtained on admission which was unremarkable for leukocytosis, hypoglycemia, or electrolyte abnormalities.

Lab parameters	Value	Reference range
Hemoglobin	12.7	13.7-17.1 g/dL
White blood cell count	9.6	4-11 k/mm^3^
Sodium	139	133-145 mmol/L
Potassium	4.2	3.6-5.2 mmol/L
Glucose	137	70-99 mg/dL
Blood urea nitrogen	15	6-20 mg/dL
Creatinine	1.2	0.7-1.2 mg/dL
Calcium	9.3	8.6-10.5 mg/dL
Magnesium	2.1	1.6-2.3 mg/dL
Alkaline phosphatase	87	40-129 U/L
Aspartate aminotransferase	14	0-38 U/L
Alanine aminotransferase	14	0-41 U/L
Troponin T	<0.01	0-0.09 ng/L
Ammonia venous	41	16-60 µmol/L
Thyroid-stimulating hormone	1.57	0.4-4 mIU/mL

## Discussion

In considering treatment for schizophrenia, both first- and second-generation antipsychotics are considered to be of equal clinical efficacy, with one exception. Clozapine, a second-generation antipsychotic, has been demonstrated to be of superior clinical efficacy in the treatment of schizophrenia. It is known to decrease mortality in treatment-resistant schizophrenia, mainly by reducing suicidal ideations [3]. However, due to its potential for severe, life-threatening side effects, clozapine is considered a second-line therapy, indicated only for patients with treatment-resistant schizophrenia/schizoaffective disorder and those with persistent self-injurious behaviors.

Clozapine has been shown to cause life-threatening neutropenia, agranulocytosis, and sedation at subtherapeutic as well as standard doses. Orthostatic hypotension, syncope, cardiac arrest, and myocarditis are additional known side effects of clozapine [4]. These adverse effects are most commonly seen during the initiation of therapy in treatment-naïve patients. As such, clozapine prescribing guidelines require doses to be titrated slowly to therapeutic levels with frequent patient monitoring. Previous cases have reported clozapine intoxication in treatment-naïve patients [1,2]. However, with regards to patients with long histories of clozapine use at standard doses with a brief period of noncompliance, reports of intoxication have not been documented. Our patient developed clozapine intoxication despite having a long history of clozapine use. The observed degree of sedation after restarting clozapine is not common in patients who have a history of previous clozapine treatment. However, we highlight the potential for adverse reactions to clozapine regardless of the duration of treatment and the necessity of tapers for all patients starting or restarting this medication.

The necessity of tapered dosing for clozapine-naïve patients is well-established. Multiple case reports have documented instances of acute intoxication in treatment-naïve patients, even at low doses [4,5]. Therapeutic dosing typically requires 300-600 mg per day; however, patients are typically started on 12.5-25 mg per day with gradual increases in dosages [6]. Little is known regarding clozapine intoxication in patients with previous clozapine treatment. Studies in pretreated patients have been limited to reports of intoxication in patients after large doses intended for suicide [2,7]. It has been suggested that previous treatment with clozapine can be protective against intoxication. Studies in animal models have supported this idea, suggesting a mechanism for clozapine tolerance [8].

Our case is unique in that the patient presented with known signs of clozapine intoxication, notably sedation, altered mental status, and dysarthria after taking therapeutic doses of the medication [2]. Clozapine metabolism varies widely between patients due to inherent metabolic differences, drug interactions, and patient characteristics such as age [1,9,10]. While evidence supports the idea of clozapine tolerance, we demonstrate the necessity of careful titration regardless of the previous treatment history. Resumption of clozapine therapy requires caution and should be restarted at low levels to avoid the possibility of acute intoxication for all patients.

## Conclusions

We highlight the potential for adverse reactions to clozapine regardless of the duration of treatment and the necessity of tapers for all patients starting or restarting this medication. While evidence supports the idea of clozapine tolerance, we demonstrate the necessity of careful titration regardless of the previous treatment history. This case highlights that when prescribing or dispensing clozapine therapy greater detail regarding prior medication compliance and treatment history should be taken into account. In addition, the resumption of clozapine therapy requires caution and should be restarted at low levels to avoid the possibility of acute intoxication for all patients.
